# Potential Effect of Intravascular Laser Irradiation of Blood (ILIB) in Improving Physical Performance: A Narrative Review

**DOI:** 10.3390/jfmk10040466

**Published:** 2025-12-01

**Authors:** Marcia Cristina Bortoleto Rotta-Ribas, Yann Zurutuza, Robson Chacon Castoldi, Paula Felippe Martinez, Silvio Assis de Oliveira-Junior

**Affiliations:** 1 Graduate Program of in Health and Development, Federal University of Mato Grosso do Sul (UFMS), Campo Grande 79070-900, MS, Brazil; marciarotta@gmail.com (M.C.B.R.-R.); zurutuza.yann@gmail.com (Y.Z.); paula.martinez@ufms.br (P.F.M.); 2 Graduate Program in Physical Exercise in Health Promotion, University North of Paraná, Londrina 86041-120, PR, Brazil; castoldi_rc@yahoo.com.br

**Keywords:** photobiomodulation, low-level laser therapy, exercise, sports performance, heart rate variability

## Abstract

**Background:** The intravascular laser irradiation of blood (ILIB) is a low-power laser technique that has been studied since the 1970s, and it is associated with the substantial capability to modulate various physiological processes. Indeed, ILIB involves the irradiation of blood with low-intensity light, typically within the red or near-infrared spectrum, to trigger a cascade of photochemical and photobiological events. **Objective:** This study aimed to analyze previous findings regarding ILIB effects on physical performance. **Methods:** This study is a narrative review of the literature, addressing the effects of ILIB on multiple organ systems and its impact on physical performance. **Results:** The most found effects include antioxidant activation, inhibition of inflammatory processes, increased blood fluidity, and improved hemorheological properties. The ILIB affects blood rheological properties based on vasodilatation and decreasing aggregation of thrombocytes. Other effects include improved deformability of erythrocytes, which results in a better supply of oxygen and a decrease in the partial pressure of carbon dioxide. Since ILIB is a photobiomodulation procedure, other applications can be considered, such as ergogenic intervention. In this context, ILIB may favor performance in aerobic exercises and contribute to practices involving anaerobic metabolism by facilitating phosphocreatine resynthesis and ATP restoration. **Conclusions:** Multiple findings seek to support the potential benefits of ILIB on metabolic and cardiovascular responses associated with exercise training, providing potential improvements in athletic performance.

## 1. Introduction

Photobiomodulation (PBM) techniques have been widely used in experimental and clinical studies for over 40 years [1,2,3,4,5,6]. Usually, PBM protocols are based on specific irradiation points to directly treat a target region using low-intensity light, such as red or near-infrared light [7,8]. These low-energy wavelengths are used because of their ability to trigger photochemical processes, like photosynthesis in plants, without causing photothermal damage to the tissue [9,10]. In humans, the current therapy resulted in healing and reduced damage by normalizing biological functions through the stimulation of beneficial processes or inhibition of harmful ones [8,9]. Indeed, PBM has been associated not only with significant biological effects on diverse tissues, such as increased cellular proliferation, accelerated healing, promotion of tissue regeneration, prevention of cell death, and anti-inflammatory activity, but also with clinical advantages such as lower pain relief [1,11,12,13,14].

Among the subtypes of PBM, intravascular laser blood irradiation (ILIB) was first characterized and used in the former Soviet Union in the 1970s [8]. Until the 1990s, emerging investigations documented that ILIB could be used to treat over several types of conditions, including disturbances associated with cardiovascular and ischemic vascular diseases, such as stroke, acute myocardial infarction, and heart failure [8,10,11]. In general, ILIB effects have been reported in almost all body systems, making this therapeutic intervention potentially helpful as a clinical intervention for various diseases [10]. ILIB is considered to induce multiple analgesic, spasmolytic, and sedative responses [7,15].

Meanwhile, the original ILIB technique involved a major disadvantage: invasive irradiation. According to Chinese references and Brazilian clinical practice, other forms of systemic and non-invasive irradiation, such as intranasal laser irradiation and cutaneous irradiation, are valid procedures [7,15]. Indeed, advances in technology and new powerful model lasers have contributed to the possibility of using the non-invasive technique, irradiating through the skin in the wrist region (the region corresponding to the radial artery), to reach blood vessels [8,16]. Currently, the ILIB technique stands out by using irradiation with systemic effects, targeting the vascular system via either a transdermal or intravascular manner. Non-invasive ILIB proceedings rely on the use of low-intensity lasers, typically in the range of 60 to 250 mW, and generally using red light (660 nm). The light emitted by the laser is monochromatic, allowing it to be precisely targeted to specific areas of the body. The treatment is non-invasive and painless, as the laser intensity is too low to cause thermal damage to the tissues [1,7,13].

In terms of scientific evidence, clinical and laboratory investigations have shown therapeutic effects of ILIB associated with the following mechanisms of action: activation of the antioxidant system, inhibition of systemic inflammatory processes, and several cardiovascular effects [1,16]. In the first case, the mechanism of action of ILIB has been associated with increased redox signaling in the respiratory chain through the stimulation of mitochondrial components, which could induce positive effects on the expression of immunoglobulins, interferons, and interleukins [10,15,17]. Indeed, some studies have shown mitochondrial changes in response to ILIB intervention, activating multiple metabolic pathways and increasing ATP production. Despite being associated with certain clinical syndromes, these alterations are not considered pathological [18,19,20].

Regarding inflammatory aspects, ILIB effects have included stimulation of both specific and nonspecific immune responses; increased levels of immunoglobulins IgG, IgM, and IgA; stimulation of interferons, interleukins, and tumor necrosis factor alpha (TNF-alpha); lymphocyte proliferation; increased phagocytic activity of macrophages; reduction in C-reactive proteins; improvements in the antioxidant enzyme system; increased regeneration of erythrocytes and microcirculation; reduced thrombocyte aggregation; activation of fibrinolysis; stimulation of nitric oxide production in monocytes with vasodilation; and improvements in endothelial dysfunction [10,18]. In the hemodynamic context, ILIB has been reported to stimulate blood circulation, triggering generalized blood irradiation effects in several systems [15,20,21]. ILIB increases the arteriovenous oxygen difference, which can eliminate tissue hypoxia and promote oxygen influx. Thus, it contributes to the normalization of tissue metabolism and improves energy supply [10].

Based on the related effects of ILIB as an intervention in chronic systemic diseases, using this procedure has been rarely reported in the literature not only to improve physical performance but also as a recovery method. Therefore, this narrative review aimed to explore and analyze findings from previous studies regarding the effects of intravascular laser blood irradiation (ILIB) on an organism. Additionally, we seek to comprehensively provide evidence for the influence of ILIB on physical performance and the cardiovascular system, particularly when this therapy is associated with physical activity practice.

## 2. Materials and Methods

### 2.1. Study Design

This study is a narrative review of the literature, addressing the effects of intravascular laser irradiation of blood (ILIB) on physical performance. A narrative literature review is a qualitative methodology that provides a background for validating premises and understanding studies, as well as stimulating reflection and controversies about a specific topic or theme from a theoretical perspective. In general, a narrative review does not necessarily require that the criteria used in the material selection process are explicit, which does not mean that researchers have not established any rules. It is useful for summarizing and synthesizing a specific area, in addition to its contribution to continuous education, being essentially qualitative [22,23].

### 2.2. Search Strategy

Comprehensive bibliographic research was conducted on the following electronic databases: National Library of Medicine (PubMed), Scientific Electronic Library Online (Scielo), Google, and Latin American and Caribbean Health Sciences Literature (LILACS). The search strategy was developed using combinations of keywords and descriptors: “photobiomodulation”; “low-level laser therapy”; “exercise”; “sports performance”; and “heart rate variability”. The inclusion criteria considered references addressing the application of ILIB in the context of health, physical exercise, sports performance, and/or heart rate variability modulation.

After the selection process, the articles were organized according to the following topics: activation of the antioxidant system: enzymatic and oxidative effects of ILIB; effects on blood cells and vascular structures; and ILIB in sports and potential applications.

## 3. Results and Discussion

### 3.1. Activation of the Antioxidant System: Enzymatic and Oxidative Effects of ILIB

The therapeutic potential of the intravascular laser irradiation of blood has received special attention, particularly regarding its impact on enzymatic activity and oxidative processes [24]. Following laser irradiation, a remarkable increase in the activity of pivotal intracellular bioenergetic enzymes was observed, highlighting the ability of this modality to modulate fundamental cellular functions. These findings suggest that ILIB may exert its effects by directly influencing the bioenergetic machinery within cells, potentially impacting metabolic pathways and cellular respiration. The capacity of HeNe laser irradiation to activate bioenergetic processes in neural mitochondria suggests that ILIB could modulate neuronal function by influencing mitochondrial respiration and ATP production within neurons [10,18]. Furthermore, the stimulation of key blood enzymes, including dehydrogenase, cytochrome-c oxidase, catalase, and acid and alkaline phosphatase, underscores the broad-spectrum enzymatic effects of ILIB, pointing towards systemic effects on multiple biochemical processes. It is plausible that the activation of these enzymes contributes to the therapeutic effects of ILIB observed in multiple clinical conditions [11,24].

Despite the application method, evidence from previous studies employing ILIB sustains a substantial biological effect, including improved mitochondrial oxidative phosphorylation, increased ATP production, reduced pro-inflammatory cytokines (e.g., IL-1β, IL-6, TNF-α) levels, and attenuated redox signaling by lowering reactive oxygen species (ROS) and lipid peroxidation markers such as malondialdehyde (MDA). These different responses reinforce systemic anti-inflammatory and analgesic effects [1]. Likewise, ILIB therapy often led to a significant increase in enzymes, quinones, and lipid peroxidation products in the bloodstream, an effect detectable within the 24 h period following each irradiation session. This initial effect on oxidative markers may represent a transient response to laser irradiation, potentially stimulating the antioxidant defense system to counteract the induced oxidative stress. Notably, there is an increase in catalase activity, with an absorption spectrum closely matching the HeNe laser wavelength, thus suggesting a direct interaction between the laser light and this crucial antioxidant enzyme [11].

**Figure 1:**
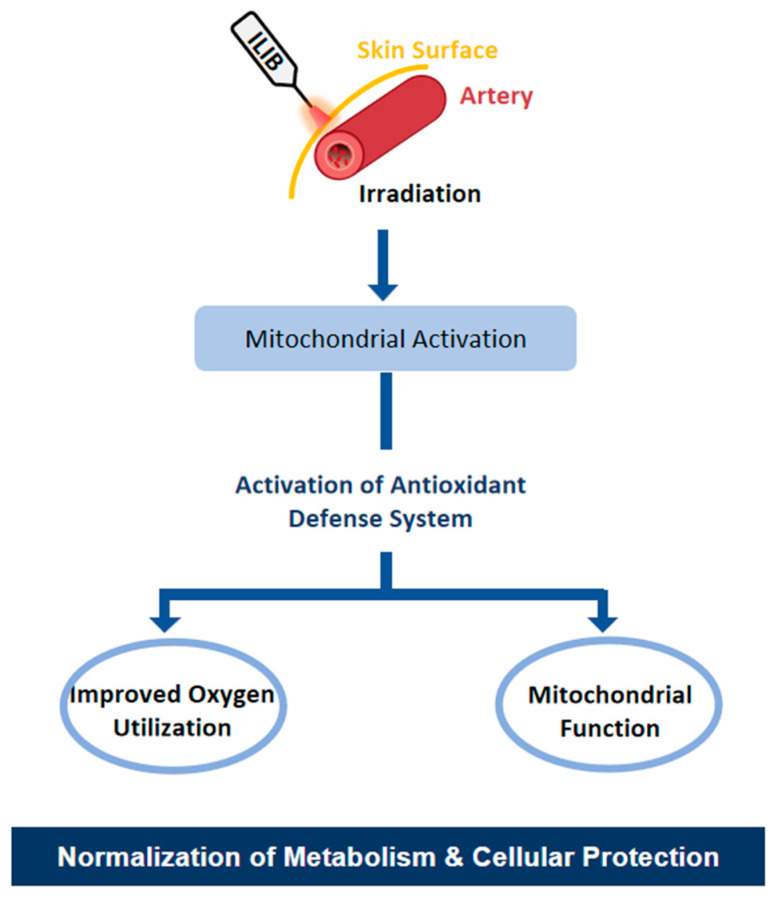
Schematic presentation of intravascular laser irradiation of blood (ILIB) effects regarding mitochondrial activity and antioxidant system; additional details may be found in previous studies [10,18,24,25,26,27].

The increase in catalase activity may represent a compensatory response to mitigate early changes in lipid peroxidation products, thereby protecting cells from oxidative damage. The enhanced oxygen transport and release rate observed with red light ILIB highlight its potential to improve tissue oxygenation [21,24]. The increase in oxygen content and the reduction in carbon dioxide particle rate following ILIB support that this therapy can not only attenuate tissue hypoxia but also enhance tissue oxygenation, potentially leading to improved cellular function and metabolic activity. These findings imply that ILIB can normalize tissue metabolism by enhancing oxygen delivery and utilization. It is worth noting that laser irradiation can induce the generation of reactive oxygen species [25]. From an immunological perspective, ILIB has demonstrated an ability to stimulate both specific and nonspecific immune responses, which enhance the production of immunoglobulins IgG, IgM, and IgA. This immunomodulatory effect may contribute to the therapeutic benefits of ILIB in conditions involving immune dysregulation. It is possible that ILIB acts as a trigger to induce immunomodulation and might minimize heavy usage of drugs and side effects, resulting in potential advantages of ILIB in terms of the levels of pain, sleep, and mood disorders [26,27] (Figure 1).

A recent study investigating the effects of ILIB on oxidative stress and energy metabolism in aged ovaries included 75 patients [27], divided into ILIB-treated and control (CTRL) groups, who underwent two cycles of laser treatment; in addition, clinical parameters were assessed. Analyses of gene expression patterns in cellular samples revealed intriguing findings in ILIB-treated patients compared with the untreated group. ILIB treatment resulted in a significant increase in the expression of the genes AIFM1 and NRF2, suggesting a potential protective effect against oxidative stress-induced cell death. Furthermore, ILIB treatment led to a downregulation of glycolysis-associated hexokinase 2 (HK2), indicating a shift away from anaerobic metabolism, along with an increase in PDHA levels, thus indicating improved mitochondrial function. Consistent with these changes, patients treated with ILIB exhibited higher expressions of key TCA cycle genes, citrate synthase (CS), succinate dehydrogenase complex subunit A (SDHA), and fumarate hydratase (FH), thus indicating improved energy metabolism. These findings reinforce the potential of ILIB as a therapeutic strategy, showing that ILIB holds promise for preserving ovarian function and reproductive health in aging individuals [27].

In turn, a recent integrative review [16] detailed potential applications of systemic photobiomodulation, focusing on two key techniques, intravascular laser irradiation of blood and vascular photobiomodulation, which can be administered intravenously or transdermally. Evidence encompassed both clinical and pre-clinical research, investigating the application of these approaches in various conditions, including respiratory, cardiovascular, metabolic, neurological, and musculoskeletal issues. Despite promising results, variability and methodological limitations of current studies on systemic photobiomodulation hinder a categorical assertion of its efficacy [16,28].

### 3.2. Effects on Blood Cells and Vascular Structures

ILIB interventions have been associated with blood adaptive alterations, including increased red blood cell count, reduced sedimentation rate, and enhanced functions of lymphatic cells and macrophages [7,24,29,30]. Likewise, experimental studies have documented substantial changes in the cellular structure of peripheral blood based on the ILIB effects [10,12]. After irradiation with the HeNe laser (632.8 nm), reticulocytosis was observed, along with an increase in red blood cell count. Increases in neutrophils (up to 192%), eosinophils (up to 111%), basophils (up to 80%), and lymphocytes (up to 19%) were also observed. All effects were verified within 1 h after the application session and included a decrease in the count of monocytes and segmented neutrophils (up to 62% and 19%, respectively). The increase in neutrophils was associated with stimulation of leukopoiesis, while a higher number of lymphocytes, basophils, and eosinophils was associated with the migration of mature cells out of the bone marrow, spleen, and lung vessels [15,24].

Previously, ILIB has been shown to decrease capillary edema and increase the number of functioning capillary tubes [10,11]. In addition, ILIB increased microcirculatory variables measured during 5 min irradiation (arteriolar pulse volume level by photoplethysmography, capillary skin blood flow by laser Doppler fluxmeter, and skin temperature by infrared thermal imaging). This response was maintained or further increased until saturation was reached during a 20 min follow-up period [24]. The increase in blood flow by ILIB is associated with the local release of nitric oxide (NO) and/or increased gene expression of NO synthases (NOS) in a variety of cellular systems. In vivo, FBM has been found to increase vasodilation in the blood vessels of mice [31] and spontaneously hypertensive rats [32].

Intravascular low-level laser irradiation elicits a cascade of adaptive responses within the circulatory system, influencing both cellular composition and vascular dynamics [24].

**Figure 2:**
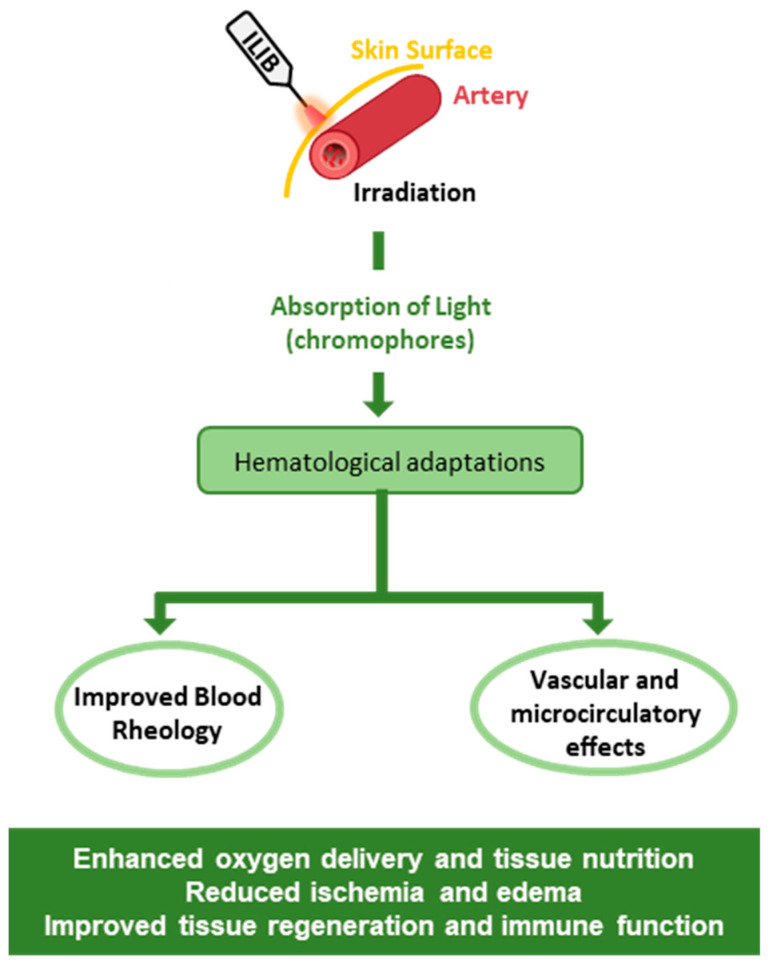
Schematic presentation of intravascular laser irradiation of blood (ILIB) effects regarding hematological and vascular adaptive alterations according to previously documented results [7,10,12,15,24,28].

Specifically, ILIB therapy may modulate the hematological profile, inducing an increase in red blood cell count and a concomitant reduction in the erythrocyte sedimentation rate, which indicates improved blood rheology [30]. Furthermore, ILIB has been shown to increase the functional capacity of key components of the immune system, such as lymphatic cells and macrophages [24,31]. These findings suggest a potential immunomodulatory role for ILIB, warranting further investigation into its effects on immune cell signaling pathways and cytokine production (Figure 2).

Experimental investigations have explored the impacts of ILIB on the cellular architecture of peripheral blood, documenting significant alterations in response to laser irradiation [26,33]. For instance, exposure to helium-neon laser light at a wavelength of 632.8 nm has been associated with an elevation in the number of circulating reticulocytes, accompanied by an increase in the overall red blood cell count. The increased presence of lymphocytes, basophils, and eosinophils could be attributed to the mobilization of mature cells from their reservoirs in the bone marrow, spleen, and pulmonary vasculature. The exact mechanisms behind these cellular changes are yet to be studied and may involve specific signaling pathways or cytokine release that controls immune cell movement [31]. Beyond its impact on cellular elements, ILIB therapy exerts notable effects on vascular structures and function. Several studies have documented the analgesic and anti-ischemic effects of ILIB, along with enhancements in blood rheology and microcirculation, especially when using a 632.8 nm low-level laser [24]. More recently, ILIB has been shown to modulate molecules from different chemical classes, although its impact on the plasma metabolome had been minimal in older women [34].

Moreover, a comprehensive systematic review investigated the diverse effects of systemic photobiomodulation in animal models, highlighting its capacity to modulate a wide range of physiological processes [28]. The main findings of the study indicated that systemic PBM positively influences blood circulation, blood flow, and blood pressure control. Additionally, the study demonstrated efficacy in reducing oxidative stress induced by hypotension, besides modulating circulating enzymatic markers in conditions such as diabetes, peripheral nerve injury, and hypercholesterolemia. There were beneficial effects on the structure of erythrocytes and the general circulatory system, as well as a significant impact on the repair processes of skin and muscle tissue [28].

Similarly, ILIB increases vascular wall permeability, improving nutrient delivery and waste removal at the tissue level, which may help treat conditions with impaired microcirculation. ILIB promotes capillary tube development, aiding in angiogenesis—the formation of new blood vessels from pre-existing ones. This process includes growth factors, endothelial cell activation, and extracellular matrix remodeling, indicating the ability of ILIB to improve microvascular function by addressing vascular spasms and stasis, as well as reversing blood flow [24]. Real-time monitoring of microcirculatory parameters during ILIB administration has yielded valuable insights into its immediate effects [31]. Previous studies show that ILIB can increase arteriolar pulse volume, cutaneous capillary blood flow, and skin temperature during a 5 min irradiation period. This response may continue or increase until reaching a saturation point within a 20 min session [33].

### 3.3. ILIB in Sports and Potential Applications

Photobiomodulation, or low-level laser therapy (LLLT), uses laser or LED light to treat various disorders and has recently gained interest [35]. LLLT aids rehabilitation by enhancing tissue growth and regeneration and reducing inflammation and pain [3,36]. In the context of sports, LLLT has become increasingly recognized for its potential to not only increase physical performance [37,38,39] but also improve post-exercise recovery [36]. Firstly, as pre-exercise interventions, photobiomodulation has been shown to increase performance and improve biochemical markers related to skeletal muscle damage and inflammation [2,6,39,40]. This therapy uses specific light wavelengths in the visible and near-infrared spectrum to influence molecular, cellular, and systemic activity [41]. Supporting tissue growth and regeneration and reducing inflammation and pain, low-level lasers have been shown to affect lymphocytes by increasing their proliferation and activation and stimulating macrophages, which enhance phagocytosis [5,31].

A pre-clinical study by Lopez et al. [4], as reviewed in Brassolatti et al. [16], investigated the capability of systemic photobiomodulation in accelerating muscle regeneration after acute muscle injury. The study applied PBM transcutaneously to the caudal vein both preventively (pre-injury) and therapeutically (post-injury), with sessions lasting 1 min and 20 s for up to 14 consecutive days. The parameters used included a 780 nm wavelength, 40 mW power, and 1.33 W/cm^2^; irradiance, totaling 3.2 J per session. The histological results revealed a reduction in inflammatory infiltrate, edema, and myonecrosis in both approaches. Notably, the preventive application demonstrated a significant decrease in serum levels of creatine kinase, aspartate aminotransferase, and lactate—important biomarkers of muscle damage—indicating a more pronounced effect with this approach. These findings suggest the potential of systemic PBM in muscle recovery and performance optimization, particularly when applied preventively [16].

**Figure 3:**
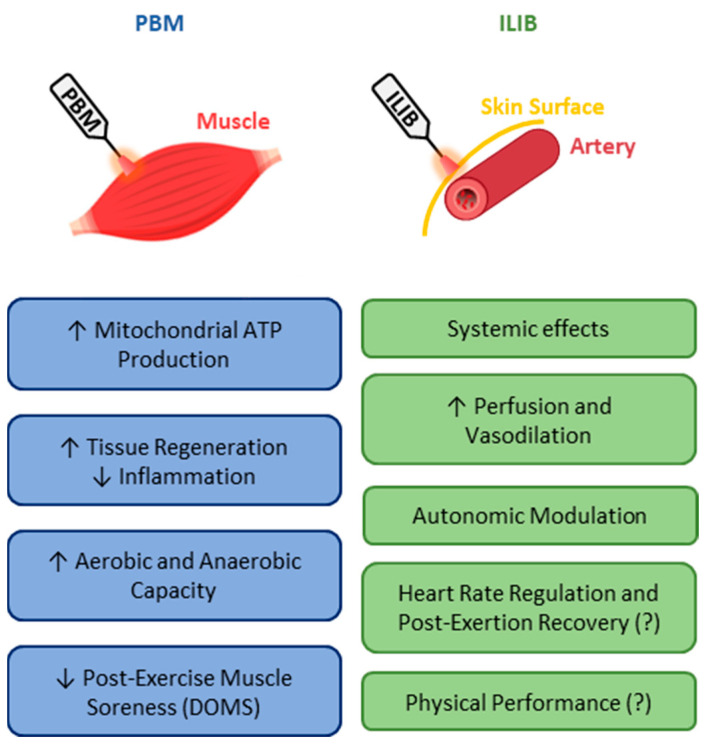
Respective and potential effects of traditional photobiomodulation (PBM) methods and intravascular laser irradiation of blood (ILIB) in sports; prepared and adapted from previous studies [11,16,18,27,31,36,37,39,43].

Since ILIB is a PBM resource, similar applications and effects could be considered, such as its use as an ergogenic or recovery aid in sports. However, when examining the use of ILIB as an ergogenic aid in sports, evidence is scarce and still in its early stages. Male bodybuilding athletes submitted to 10 ILIB application sessions showed increased maximum strength and muscular endurance in response to a 16-week ILIB protocol [42]. Based on the different effects associated with ILIB, improving performance is a potential effect of producing multiple cellular events that could increase skeletal muscle function and post-exercise recovery. ILIB may favor performance in aerobic exercises and contribute to exercises involving anaerobic metabolism, facilitating phosphocreatine resynthesis and ATP restoration (Figure 3).

Further research should focus on elucidating the optimal parameters of ILIB protocols in athletes, as variations in light sources, wavelengths, power outputs, and exposure durations can affect different outcomes. Additionally, further studies should aim to improve our understanding of the therapeutic capacity of irradiation lasers.

Based on the provided information regarding the effects of intravascular blood irradiation (ILIB), several hypotheses can be elaborated on how this therapeutic intervention may affect anaerobic and/or aerobic metabolism. Indeed, ILIB may modulate energetic metabolism due to its effects on ATP production and metabolic regulation [18,24]. As ILIB stimulates the activation of the antioxidant system [27] and promotes greater redox signaling in the respiratory chain [8,15], it could increase the availability of ATP during exercise training. In this perspective, the activation of metabolic pathways associated with mitochondrial pathways [10] could result in more efficient energy production, potentially enhancing performance in short-duration events, especially during intense demands such as weightlifting and sprints.

In terms of aerobic metabolism, ILIB could result in a significant impact due to its effects on blood circulation and oxygen delivery to tissues. ILIB stimulates blood circulation and increases oxygen delivery to muscles during aerobic exercise training [11,24]. This can lead to greater aerobic capacity and higher muscle endurance, allowing athletes to sustain prolonged efforts more efficiently. Additionally, the reduction in tissue hypoxia promoted by ILIB may contribute to the normalization of aerobic metabolism [10], allowing a more effective utilization of energy substrates during prolonged effort demands. As a result, athletes may experience increased aerobic capacity, allowing them to maintain higher levels of performance over extended periods. The potential reduction in muscle fatigue and enhanced endurance could be particularly beneficial for endurance athletes, such as long-distance runners or cyclists.

Based on the effects mentioned, a hypothesis can be formulated suggesting that ILIB may improve muscle recovery. Given their reported effects on stimulating blood circulation, not only promoting antioxidant activation [27] but also reducing inflammation [4], ILIB could improve muscle recovery following strenuous exercise. By increasing blood flow to the muscles, ILIB may facilitate the removal of metabolic products and provide essential nutrients and oxygen for tissue repair, sustaining similar effects from different recovery methods, such as cryotherapy [44,45,46,47] or massage [48,49,50]. Further methods of recovery, including physiological (types of active recovery, sleep, and rest) and nutritional interventions, have also been proven useful [51,52]. Consumption of green tea, beetroot gels, creatine, or alkaline water supplementation improves recovery processes [51]. Since they consist of restoring body systems to baseline conditions, providing physiological equilibrium and preventing injury onset [53,54,55], effects from potential combinations of ILIB with these different methods are yet to be investigated. They could represent advantageous options that accelerate muscle recovery and improve performance following fatigue onset, especially at higher sport demand levels.

In summary, ILIB may influence both anaerobic and aerobic metabolism, offering a range of potential benefits for athletic performance. ILIB could increase the availability of ATP by stimulating metabolic pathways, particularly by improving mitochondrial performance and in the resynthesis of ATP, leading to enhanced energy efficiency. As a result of increased availability of ATP, athletes could sustain peak performance for longer periods, ultimately contributing to improved overall performance. In this context, this effect could be beneficial not only during high-intensity and short-duration activities but also in endurance exercises. Since the use of ILIB as an ergogenic resource in sport is still scarce, clarifying comparative effects on different sport modalities should be further investigated.

## 4. Conclusions and Future Directions

Considering several interesting effects of ILIB as an intervention in chronic systemic diseases, which were documented in previous studies, further research is needed to clarify potential effects in physically active individuals. Indeed, addressing this procedure not only to improve physical performance but also as a recovery method has yet to be better exploited in sports sciences. Indeed, if it is possible to replicate the effects of ILIB on active individuals, it cannot be ruled out that physical performance could be improved. With the activation of the antioxidant system, an important tool is available for minimizing the deleterious effects of physical exercise, especially regarding the production of reactive oxygen species (ROS), which can cause cellular and tissue damage. Furthermore, ILIB may be advantageous for physical performance by inhibiting systemic inflammatory processes and minimizing fatigue. Among other effects, increased blood fluidity can improve the delivery of oxygen and nutrients to the muscles during physical exercise, increasing the capacity of the body to produce energy and perform physical activities more efficiently.

Consequently, there are several promising directions for further research. To advance in this direction, it is crucial to develop a multidisciplinary and collaborative approach between sports medicine professionals, exercise physiologists, biochemists, and photobiomodulation specialists. Within this perspective, there are multiple possibilities for investigation, and they are as follows:

- Studies in Active Athletes: To conduct specific studies in athletes from different sports to assess the effects of ILIB on physically active individuals, including analyses of physical performance, recovery capacity, fatigue resistance, and improvements in muscle function.
- Post-Training Recovery: To investigate how ILIB can accelerate the post-exercise recovery process in athletes, including reducing delayed onset muscle soreness, restoring energy levels, and restoring metabolic balance.
- Impact on Sports Injuries: To exploit the potential of ILIB in the prevention and rehabilitation of sports injuries, including muscle, tendon, and articulation injuries. Longitudinal studies can help us understand whether ILIB therapy can reduce recovery time and promote faster healing.
- Analysis of Physiological Mechanisms: To investigate the physiological mechanisms underlying the effects of ILIB on sports performance, including its impact on muscle metabolism, inflammatory response, and antioxidant system.
- Exploration of ILIB Therapy Protocols Based on Individual Needs: To explore the potential of ILIB in specific populations such as adolescents [16], older adults, or individuals with chronic conditions [1,14].
- Interaction with Other Interventions: To investigate the synergistic effects of ILIB when combined with other interventions, such as resistance training, sports nutrition, and recovery techniques, aiming to optimize athletic performance.
- Long-Term Studies: To assess the long-term effects of ILIB on sports performance, as well as its long-term safety and tolerability in elite and recreational athletes.

By addressing these future directions, science may provide valuable insights into the potential of ILIB as a promising therapeutic resource to improve sports performance and recovery, promoting benefits to health and well-being for athletes. Indeed, the ILIB technique is considered a safe intervention method for preventive application and/or treatment, with minimal risks for patients. In terms of ethical and safety contexts, a recent narrative review evaluated the clinical efficacy and safety of ILIB in multiple medical conditions based on randomized clinical trials. The study highlighted that ILIB can provide significant systemic benefits, including pain reduction, inflammation control, and functional improvements, such as improved quality of life and systemic metabolic function. Notably, ILIB proved to be safe and well-tolerated, posing a low risk of adverse effects [35].

Nevertheless, it is worth mentioning that methodological limitations common to published studies, such as failed standardization in the reported ILIB parameters and/or absence of information across diverse investigations, have compromised discussions about potential clinical applications according to previous reviews [1,16]. The current limitations impair comparisons among different application protocols in terms of used parameters, making it difficult to correlate their benefits in clinical practice. Therefore, the development of new investigations is critical and necessary to clarify not only mechanisms of action but also to show the efficacy of ILIB based on well-detailed methodologies.

## Data Availability

No new data were created or analyzed in this study. Data sharing is not applicable to this article.
